# Supplementary figures and images for: A Vision-Based Sensing Approach for a Spherical Soft Robotic Arm
Source: Front Robot AI. 2021 Feb 26;8:630935. doi: 10.3389/frobt.2021.630935 (PMC7953419; doi:10.3389/frobt.2021.630935)

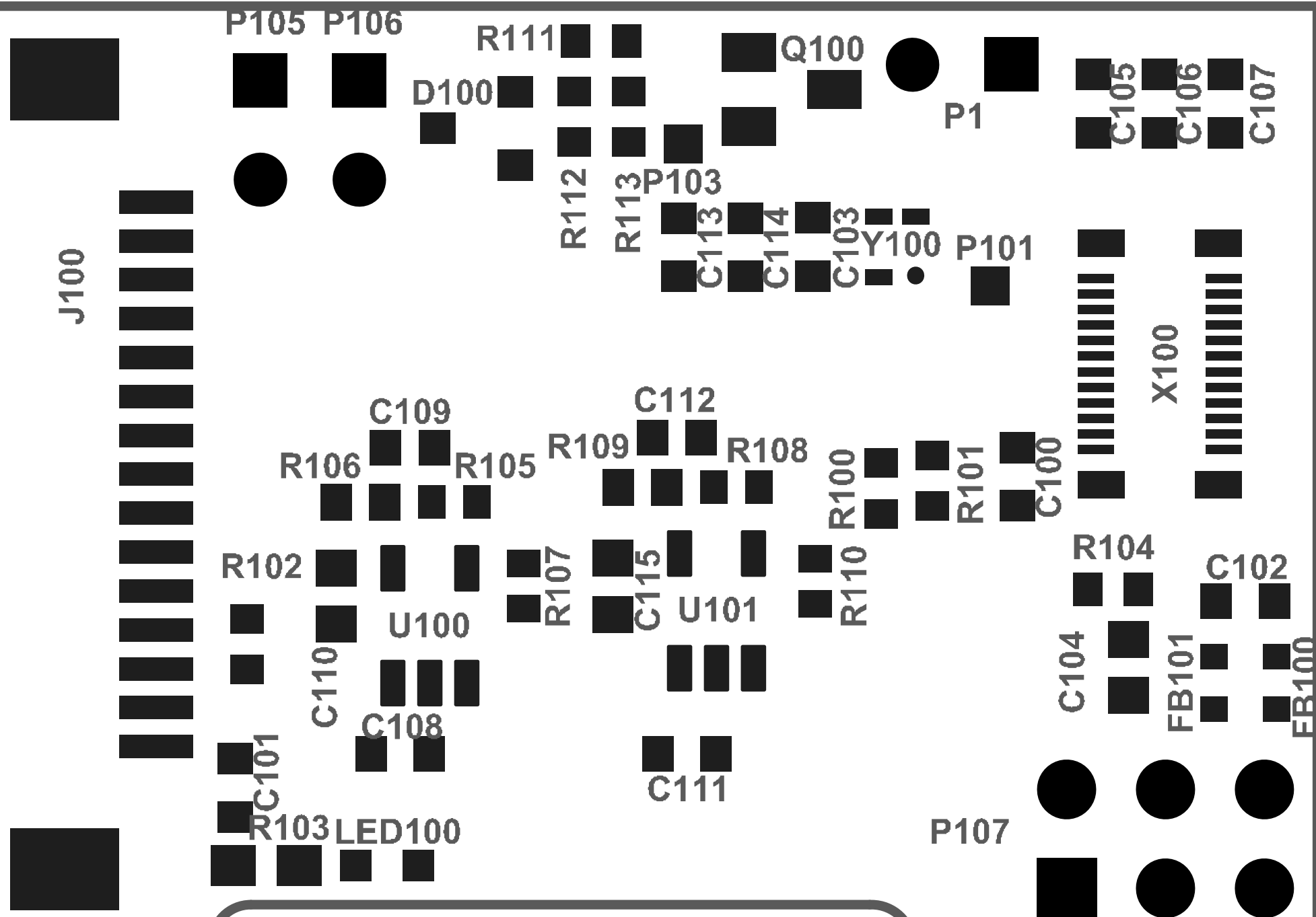

Supplement: Supplementary file 1 [file datasheet1.zip › supplementaryFiles/Electronics/ArducamAdapter_CameraBoard_Rev01/PCBA/Assembly Drawing.pdf]

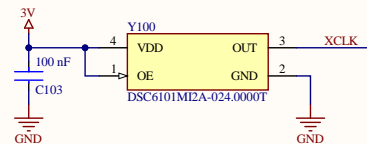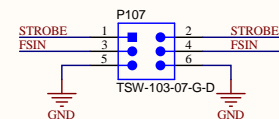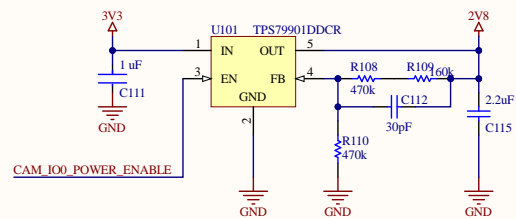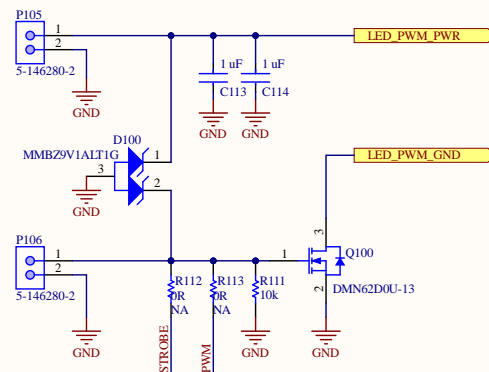

|            |                           |           |          |
|------------|---------------------------|-----------|----------|
| Title      |                           |           |          |
| Size<br>A3 | Number                    |           | Revision |
| Date:      | 6.25.2020                 | Sheet of  |          |
| File:      | C:\Projects\Camera.SchDoc | Drawn By: |          |

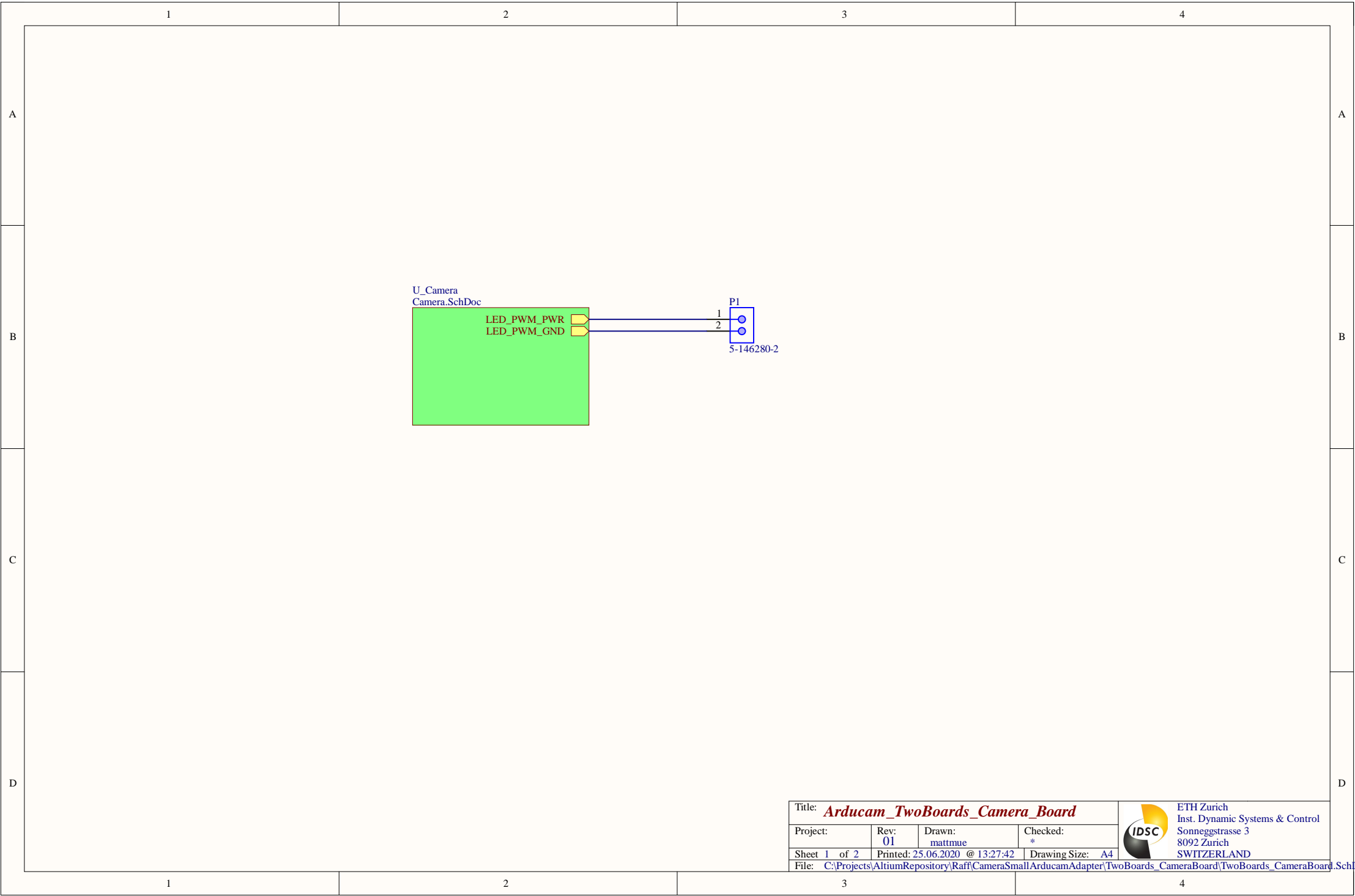

Supplement: Supplementary file 1 [file datasheet1.zip › supplementaryFiles/Electronics/ArducamAdapter_CameraBoard_Rev01/PCBA/TwoBoards_CameraBoard.pdf]

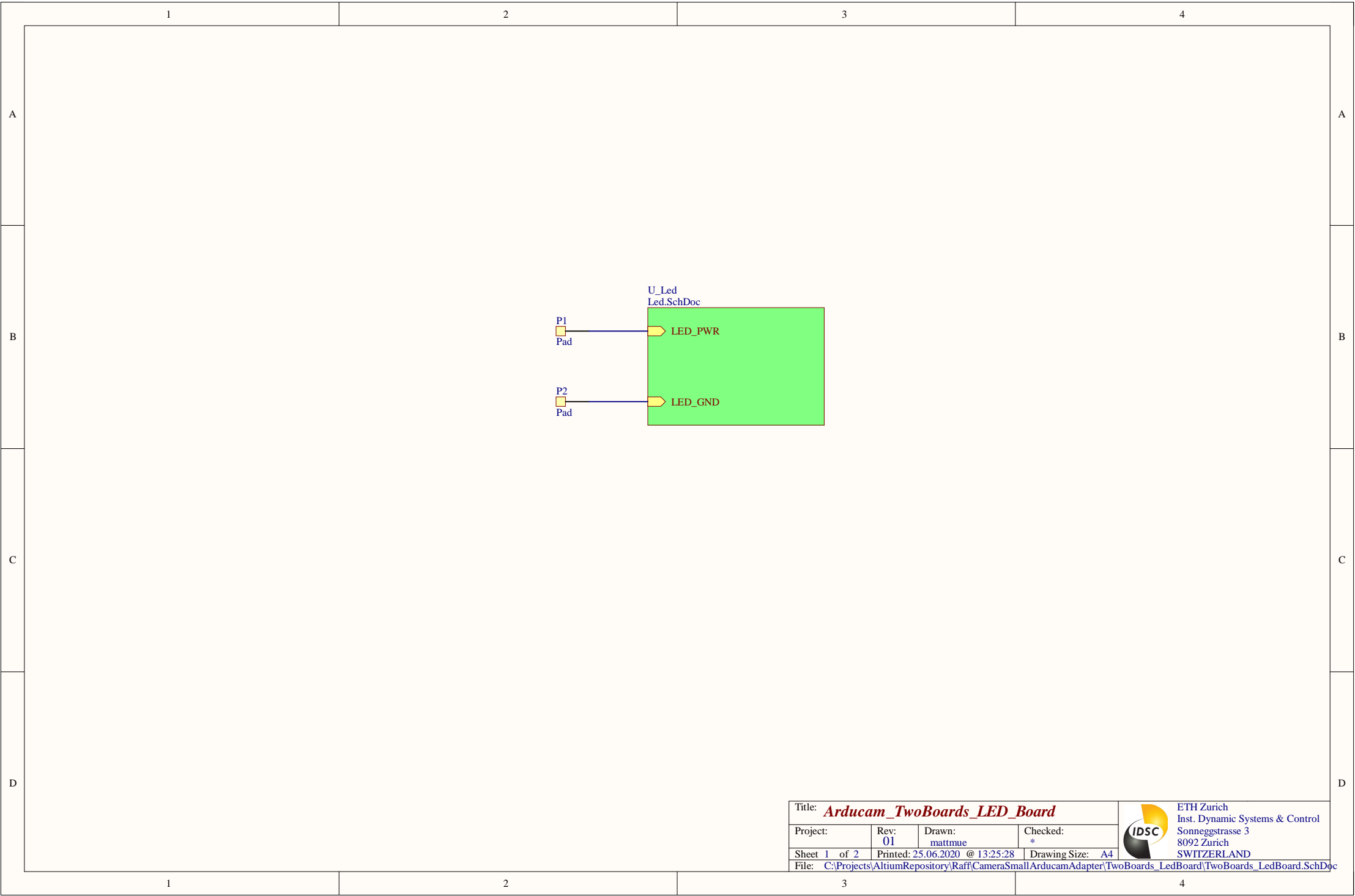

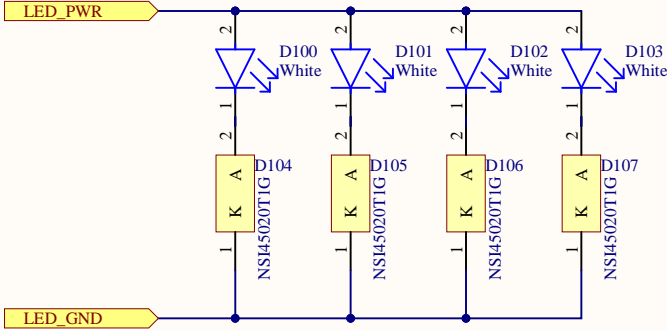

|            |                            |           |
|------------|----------------------------|-----------|
| Title      |                            |           |
| Size<br>A4 | Number                     | Revision  |
| Date:      | 6.25.2020                  | Sheet of  |
| File:      | C:\Projects\...\Led.SchDoc | Drawn By: |

Supplement: Supplementary file 1 [file datasheet1.zip › supplementaryFiles/Electronics/ArducamAdapter_LEDBoard_Rev01/TwoBoards_LedBoard.pdf]
